# Psi-Caller: A Lightweight Short Read-Based Variant Caller With High Speed and Accuracy

**DOI:** 10.3389/fcell.2021.731424

**Published:** 2021-08-13

**Authors:** Yadong Liu, Tao Jiang, Yan Gao, Bo Liu, Tianyi Zang, Yadong Wang

**Affiliations:** Center for Bioinformatics, Faculty of Computing, Harbin Institute of Technology, Harbin, China

**Keywords:** variant calling, partial order alignment, short read sequencing, SNV/indel detection, local assembly

## Abstract

With the rapid development of short-read sequencing technologies, many population-scale resequencing studies have been carried out to study the associations between human genome variants and various phenotypes in recent years. Variant calling is one of the core bioinformatics tasks in such studies to comprehensively discover genomic variants in sequenced samples. Many efforts have been made to develop short read-based variant calling approaches; however, state-of-the-art tools are still computationally expensive. Meanwhile, cutting-edge genomics studies also have higher requirements on the yields of variant calling. Herein, we propose Partial-Order Alignment-based single nucleotide polymorphism (SNV) and Indel caller (Psi-caller), a lightweight variant calling algorithm that simultaneously achieves high performance and yield. Mainly, Psi-caller recognizes and divides the candidate variant site into three categories according to the complexity and location of the signatures and employs various methods including binomial model, partial-order alignment, and de Bruijn graph-based local assembly to handle various categories of candidate variant sites to call and genotype SNVs/Indels, respectively. Benchmarks on simulated and real short-read sequencing data sets demonstrate that Psi-caller is times faster than state-of-the-art tools with higher or equal sensitivity and accuracy. It has the potential to well handle large-scale data sets in cutting-edge genomics studies.

## Introduction

High-throughput sequencing (HTS) has become a fundamental approach to characterize human genomes (Lander et al., 2001; Shendure et al., 2019). Especially, the discovery of genomic variants from HTS data, i.e., variant calling, is one of the most important HTS applications that is fundamental in many genomics studies to discover the associations between genome variations and important phenotypes and diseases (Shastry, 2002; Weischenfeldt et al., 2013; Kosugi et al., 2019), as well as the diversity and evolution of human genomes at both individual and population levels (Auton et al., 2015; Wu et al., 2019). Single nucleotide polymorphisms (SNVs) and short insertions/deletions (Indels) are the genomic alteration that usually refers to the change of less than 50-base pair (bp) nucleotide fragments compared to structural variants. Variant calling is the detection of the nucleotide differences between donor and reference genomes from millions to billions of HTS reads. With the rapid development of HTS technologies such as Illumina platform (Caporaso et al., 2012) and long-read sequencing technologies, such as Pacific Biosciences (PacBio) (Roberts et al., 2013) and Oxford Nanopore Technologies (ONT) (Jain et al., 2016), the calling of variants are promising with high yield and precision. Long reads greatly improve the detection of structural variants because of the long-range spanning information (Goodwin et al., 2016). However, long reads often suffer from high error rates including substitution, small insertions, and deletion (Roberts et al., 2013; Jain et al., 2016), which is still non-trivial for long read-based caller to distinguish genuine variants and sequencing errors. For most short-read sequencing platforms, Indel errors are rare and simultaneously achieve high base accuracy (>99%), which have been proven more helpful for SNV/Indel calling in several large-population genome projects (Auton et al., 2015; Wu et al., 2019).

However, existing variant calling approaches are computationally expensive and/or have relatively low sensitivity and accuracy, which becomes the bottleneck of variant calling for large-scale studies, such as population sequencing projects, which requires analyzing tens of thousands of data sets. It is on demand to develop more advanced bioinformatics approaches with higher speed, sensitivity, accuracy, and scalability. In recent years, many efforts have been made to develop alignment-based approaches such as GATK (McKenna et al., 2010), FreeBayes (Garrison and Marth, 2012), and Clair (Luo et al., 2020), which has been mainstream approaches in short read-based variant calling. Such approaches extract signatures of SNVs and Indels from the pileups of the reads in relatively small genomic regions, and various kinds of methods, such as local assembly (GATK), Bayesian statistics (FreeBayes, GAKT), and deep neural network (Clair), are used to analyze the features to detect potential variants. In detail, GATK uses logistic regression to model base errors, hidden Markov models to compute read likelihoods, and naive Bayes classification to identify variants, which are then filtered to remove likely false positives using a Gaussian mixture model with hand-crafted features capturing common error modes. FreeBayes enables direct detection of haplotypes from short reads using a Bayesian statistical framework, which is capable of modeling multiallelic loci in sets of individuals with non-uniform copy number. Clair supports variant calling for short reads and long reads using a deep learning-based method, where the summary of aligned reads around putative candidate variant sites was used as input of the deep learning framework.

Herein, we propose Partial-Order Alignment-based SNV and Indel caller (Psi-caller), a novel ultrafast and versatile alignment-based variant calling approach with two key features. (1) Psi-caller recognizes candidate variant sites from pileup alignments and then divides the sites into three different categories according to the complexity and position of signatures. (2) Psi-caller applies various approaches, i.e., binomial model, partial-order alignment (POA) and de Bruijn graph-based local assembly, to detect and genotype SNVs and Indels in the above three different categories. The speed of Psi-caller is faster than that of state-of-the-art variant callers, especially one order of magnitude faster than some of the most popular approaches such as GATK and FreeBayes. Moreover, it also has higher or equal yields as well. We believe that it has the potential to play an important role in many forthcoming genomic studies.

## Methods

### Overview of the Psi-Caller Approach

**FIGURE 1:**
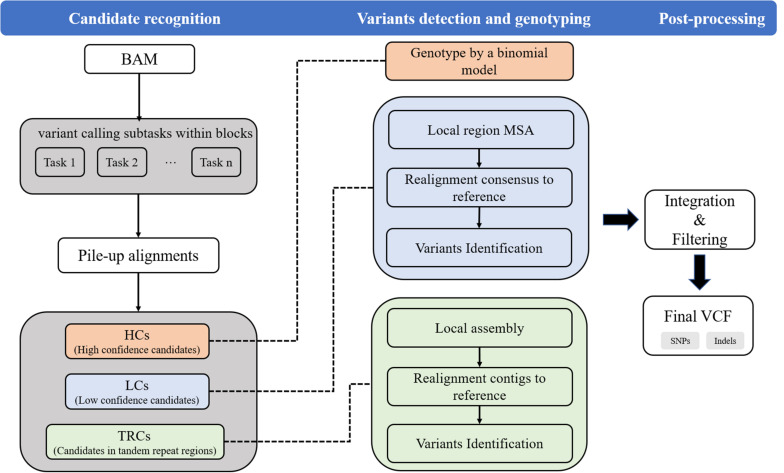
The workflow of Psi-caller approach. Psi-caller detects SNV/Indels mainly in three steps. In step 1 (“Candidate recognition”), Psi-caller first generates multiple tasks according to chromosome and block size for parallelization and then extract candidate variant site separately for each task. In step 2 (“Variants detection and genotyping”), Psi-caller implements three difference approaches to generate variant calls and assigns genotypes. In step 3 (“Post-processing”), Psi-caller concatenates the resulting output from various tasks and removes duplicated variants.

Psi-caller uses sorted BAM or CRAM files as inputs. The input files can be provided by commonly used read aligners such as BWA (Li and Durbin, 2009). Mainly, Psi-caller extracts the divergences between reads and reference from the detailed information of read alignments (i.e., CIGARs) and uses them as signatures to detect and genotype SNVs and short Indels. The approach has four major steps as follows, and a schematic illustration is shown in Figure 1.

(1) Task splitting: Psi-caller splits reference genome into fixed-size blocks to make a number of variant calling subtasks. The subtasks are further operated in a parallel way with multiple CPU threads.
(2) Candidate recognition: Psi-caller analyzes the divergences between the reads and reference to recognize candidate SNV and Indel sites. Local genomic regions with variant signatures are recognized as candidates and categorized into three classes according to their positions and the ratio of supporting reads, i.e., high confidence candidates (termed as HCs), low confidence candidates (termed as LCs), and candidates in tandem repeat regions and low-complexity regions (termed as TRCs).
(3) Variants detection and genotyping: Psi-caller employs various approaches, i.e., direct binomial model, POA, and de Bruijn graph-based local assembly, to call and genotype SNVs and Indels in HCs, LCs, and TRCs.
(4) Post-processing: Psi-caller integrates the variants from multiple subtasks and filters the calls with relatively low scores.

### Task Splitting

Psi-caller divides the input data into blocks to implement parallel computing. Benchmarks on several data sets in various sizes suggest that this strategy greatly helps achieve highly scaling performance (refer to “Results” section for details). In detail, a user-defined size parameter is employed (default: 20,000,000 bp) to split the reference genome non-overlapping blocks. For each block, a Psi-caller instance (termed as a task) is run and output the corresponding results to a separate intermediate file. Thus, all the tasks are run in a parallel way using GNU parallel (Tange, 2020) with a user-defined number of CPU cores.

### Candidate Variant Sites Generation

For each task, Psi-caller uses Samtools (Li et al., 2009) to extract detailed information of read alignment, including the chromosome, starting position, and CIGARs for each of the reads. All the alignments with low quality (default: MAPQ < 10, can be tuned with “-q” option) and supplementary alignments (flag = 2048), secondary alignments (flag = 256), mate unmapped reads (flag = 8), and unmapped reads (flag = 4) are filtered out. The remaining high-quality alignments are then traversed from upstream to downstream to make a triple consisting of three variables, i.e., (*V*,*S*,*L*), which can be seen as a summary of the pileup read alignments. A schematic illustration is shown in Supplementary Figure 1.

(1) *V* (Dictionary) lists the number of alternate bases at a SNV, INDEL, or the number of reference base otherwise. For example,

$$V=\begin{array}{c} \{"A":C_{1},"C":C_{2},"G":C_{3},"T":C_{4},"N":C_{5},"I": \end{array}\begin{array}{c} C_{6},"D":C_{7}\} \end{array}.$$

(2) *S* (Integer) is the accumulated base quality for alternative bases.
(3) *L* (Dictionary) lists the inserted or deleted sequence. For example,

L={"⁢I⁢":["⁢AC⁢","⁢AC⁢"],"⁢D⁢":["⁢TTA⁢","⁢TA⁢"]}.

Psi-caller sorts *V* according the number of base occurrences and sums all the occurrences as *T*. Psi-caller calculates the frequencies of various alternative bases at each genomic position by the following equation:

(1) $$\begin{array}{c} P_{a\,l\,t}=c_{a\,l\,t}/T \end{array}$$

where *c*_*alt*_ is the number of a specific kind of alternative base listed in *V*. A position is recognized as a candidate variant site if there is at least one kind of alternative base whose frequency is higher than a threshold *T*_*alt*_. It is also worth noting that there could be multiple alternative bases having frequencies higher than *T*_*alt*_. In this situation, Psi-caller records all of them and recognizes this site as a multi-allele site. In order to reduce the false positives caused by sequencing errors or mistakes in alignments, Psi-caller calculates the average base quality of all the alternative bases at this genomic position by *BQ*_*ave*_ = *S*/*N*, where *N* is the total number of alternative bases at this genomic position. The position is discarded if *BQ*_*ave*_ < 20 empirically.

The simple rules mentioned above is able to detect a large proportion of variants since they have homogeneous and evident signatures, an example is shown in Supplementary Figure 2. However, there are a small proportion of variants with more complex signatures, which are usually longer Indels and/or located in repeat-rich regions; an example is provided in Supplementary Figure 3. There are usually relatively poor read alignments around the variant sites, and more advance methods are needed. To address these issues, Psi-caller divides all the candidate variant sites into HCs, LCs, and TRCs beforehand according to the complexity of the signatures as well as genomic locations to handle them in various approaches.

Herein, a candidate variant site is regarded as a HC if it meets the following conditions:

(1) It has high supporting evidences, in practice, *P*_*alt*_ > 0.5.
(2) The location of the site is out of tandem repeat and low-complexity regions (LCRs).
(3) There is no other candidate variant site within flanking sites (100 bp upstream and downstream empirically).

A candidate variant site is regarded as an LC if it satisfies the following conditions:

(1) The highest frequency of all the alternative bases is less than 0.5, or it is a multi-allele site.
(2) The location of the site is out of tandem repeat and low-complexity regions (LCRs).
(3) There is another candidate variant site within 100 bp upstream or downstream. It is worth noting that a candidate variant site is categorized as an LC if this condition is met, even if it has a >0.5 frequency for any alternative base.

Sequencing-based methods of variant detection can be confounded by repetitive and low-complexity regions (LCRs) (Trost et al., 2018). The LCRs contains (1) assembly gaps that includes centromeres, telomeres, and constitutive heterochromatin domains; (2) segmental duplications; (3) the pseudo-autosomal regions of the sex chromosomes. The short tandem repeats are defined by RepeatMasker (Smit et al., 2015).

A candidate variant site is regarded as a TRCs if it is located in short tandem repeat regions or LCRs. Herein, Psi-caller supports the use of an annotation (in BED format) on short tandem repeat and LCRs for reference genome to mark such regions in advance. In this study, the employed repeat annotation on human reference genome is available at https://github.com/PacificBiosciences/pbsv/tree/master/annotations, and the CLR annotation on human reference genome is available at http://tcag.ca/documents/projects/RLCRs_no_Repeat_Masker.zip.

For Indels, *L* records the allele sequence for all supporting alignments. If the context and length of allele sequence are divergent, the Indel candidate variant site is also regarded as LC.

### Variant Detection and Genotyping

Psi-caller handles various types of candidate sites with three different approaches: (1) for HCs, Psi-caller employs a binomial model with a genotype prior probability to call and genotype variants; (2) for LCs, Psi-caller uses a SIMD-based fast POA method proposed in our previous study [abPOA (Gao et al., 2020)] to generate the consensus sequence of the reads of the potential variant sites; and (3) for TRCs, Psi-caller uses a de Bruijn graph-based local assembly on a larger local region to generate contigs of the reads. The POA-based and local assembly based methods enable high-quality consensus sequences of the reads around variant sites to be generated, and the variants are detected by realigning the consensus sequences against local reference. Moreover, the assembly based method has higher ability to handle local short repeats.

#### Variant Calling With Binomial Model for HCs

For an HC site, Psi-caller simply uses the alternative base as alternative allele if it is a single-allele site. The corresponding genotype is inferred by a maximum likelihood strategy where the likelihoods of three possible genotypes are computed by a binomial model, assuming a diploid individual

(2) $$\begin{array}{c} \left\{ \begin{array}{c} L\,\left(\frac{0}{0}\right)=\frac{1-p\,r\,i\,o\,r}{2}\times {\left(1-\varepsilon \right)}^{S\,R_{R\,e\,f}}\times \varepsilon^{S\,R_{A\,l\,t}}\\ L\,\left(\frac{0}{1}\right)=p\,r\,i\,o\,r\times {\left(\frac{1}{2}\right)}^{S\,R_{R\,e\,f}+S\,R_{A\,l\,t}}\\ L\,\left(\frac{1}{1}\right)=\frac{1-p\,r\,i\,o\,r}{2}\times {\left(1-\varepsilon \right)}^{S\,R_{A\,l\,t}}\times \varepsilon^{S\,R_{R\,e\,f}} \end{array}\right. \end{array}$$

where ε is the probability of a read being mapped to a given allele mistakenly (default: 0.03), assuming it is constant and independent between all observations; *prior* is the prior probability for a heterozygous genotype. Herein, the prior distribution is configured as equal for all the three genotypes, i.e., *prior* = 1/3, which was determined by empirical experiments; *SR*_*Ref*_ and *SR*_*Alt*_ are the numbers of the read alignments carrying reference and alternative alleles, respectively.

#### Variant Calling With SIMD-Based POA Approach for LCs

For an LC site, a more advanced POA-based approach is used since the binomial model produces more false positives, mainly caused by the more complicated read alignments. Variant calling is implemented in the following four steps:

(1) Psi-caller traverses and merges the low confidence sites from upstream to downstream. An LC site is merged by an upstream site if their distance is shorter than a predefined threshold *T*_*lc*_ (default value: 10 bp). Psi-caller considers a set of merged LC sites as a new low confidence region and extends it both upstream and downstream with *f* bp (default value: 25 bp).
(2) For the extended region, Psi-caller extracts all the spanning and overlapping alignments. Alignments with overlapped ratio less than 0.5 are discarded. Further, Psi-caller employs abPOA to generate one or more consensus sequences of the involved read parts.
(3) Psi-caller aligns the generated consensus sequence(s) against the reference sequence of the candidate region (by KSW2 (Li, 2018; Suzuki and Kasahara, 2018)) and recognizes alternative allele(s) from the detailed alignment information. Moreover, it also records the numbers of supporting reads to the reference and alternative allele(s).
(4) If the base quality of the reads is not available, Psi-caller uses the binomial model same to that of high confidence candidate variant site. Otherwise, Psi-caller implements an all-to-all alignment for all the read parts and the reference and alternative alleles to calculate the likelihoods of various alleles. Further, a Bayesian model (refer to “The Bayesian approach”) is used for computing the probability of various genotypes.

To improve the accuracy of consensus sequence(s) in the second step, Psi-caller clips the whole alignments at the starting and ending positions to get homogeneous breakpoint.

#### Variant Calling With Local Reassembly Approach for TRCs

For repeat-rich regions like tandem repeats and segmental duplications, read mapping-based methods usually fail to give reliable variant calls because of the difficulties of the accurate alignment between short reads and local repeats. The de Bruijn graph has been widely used and efficient for representation of genomic repeats as a repeat graph by short-read assembly approaches and variant callers. Thus, for a TRC site, Psi-caller implements a four-step local assembly based method to handle the repeats and reference bias to make more accurate calls as follows:

(1) Psi-caller traverses all the TRCs and combines candidate variant sites into roughly 300-bp reassembly windows.
(2) For each reassembly window, all the overlapped reads are used to construct a de Bruijn graph. Increasing length of the k-mers (from 41 to 75, increased by 5) are flagged and treated separately to avoid loops in the de Bruijn graph. The generated contigs from previous iteration are set as pseudoreads for next-round contig generation. One or more haplotypes (default value: 2) are derived from the de Bruijn-like graph using depth-first search by prioritizing variants with the highest degree of read support and scored.
(3) KSW2 is then used to realign the candidate haplotypes to local reference sequence and recognize variants from the alignment.
(4) The inferring of genotype is the same to that of LCs.

#### The Bayesian Approach

To genotype a specific variant site in the donor genome, we apply a simple Bayesian statistic relating *Pr*(*G*_*i*_|*R*_*i*_) to the likelihood of sequencing errors in the reads used for POA or local assembly and the prior likelihood of specific genotypes. For a given genotype, we use simple Bayes’ rule to relate the probability of a specific genotype quality of sequencing observations and with prior expectations as follows:

(3) $$\begin{array}{c} P\,r\,\left\{G|R\right\}=\frac{P\,r\,\left\{G\right\}\cdot P\,r\,\left\{R|G\right\}}{P\,r\,\left\{R\right\}}=\frac{P\,r\,\left\{G\right\}\cdot P\,r\,\left\{R|G\right\}}{\sum_{i}P\,r\,\left\{G_{i}\right\}\cdot P\,r\,\left\{R|G_{i}\right\}} \end{array}$$

where, *Pr*(*R*_*i*_|*G*_*i*_) is the probability of a sample genotype given sequencing observations, which is represented by the following.

(4) $$\begin{array}{c} \text{Pr}\,\left\{R|G\right\}=\prod_{j}\left(\frac{P\,r\,\left\{R_{j}|H_{1}\right\}}{2}+\frac{P\,r\,\left\{R_{j}|H_{2}\right\}}{2}\right) \end{array}$$

Assuming a diploid sample, *H*_*1*_ and *H*_*2*_ are two alternative alleles on haplotypes, respectively. *Pr*(*R*|*H*) is the haploid likelihood function, which can be related to a specific combination of errors of sequencing observations. In practice, Psi-caller aligns local reads to the consensus sequences and contigs to collect the mismatches, Indels nearby, and then *Pr*(*R*|*H*) can be calculated by the local mapping quality scores.

(5) $$\begin{array}{c} P\,r\,\left(R|H\right)=10^{-\sum_{e}\frac{Q_{e}}{10.0}} \end{array}$$

assuming there are *e* errors and they are independent at different sites of the read. *Q_e* is the Phred-scale base quality.

The Bayesian model is applied to compute the probability of various genotypes after POA of local assembly by implementing an all-to-all alignment for all the read parts and the reference and alternative alleles when the base quality of the reads is available.

### Variant Integration and Filtering

Psi-caller concatenates all the results (in VCF format) from various tasks using *vcfcat* and then removes duplicated variants. If a variant detected by an HC site can also be recognized from a POA-based or assembly based result from another task, Psi-caller chooses the one with higher variant quality as a final result.

## Materials

### Simulated Data Sets for Benchmark

Using the curated benchmark sets for single-nucleotide (SNV), small insertion and deletion (IDNEL) of HG001 sample from the Genome in a Bottle (GIAB) consortium as ground truth, 269,554 SNVs and 42,136 Indels in chromosome 2 of human reference genome (version: hs37d5) were selected to generate simulated data sets and used to assess the “baseline” sensitivity and accuracy of Psi-caller. In the simulation study, two donor haplotypes carrying all the “homozygous” and “heterozygous” variants were generated, and then 40× Illumina-like data sets were simulated using the following three steps:

(1) Variant with genotypes equal to “0| 1” or “1| 1” were extracted to generate the VCF file containing all variants in the first *in silico* haplotype (termed as Hap1). Similarly, variants with “1| 0” and “1| 1” genotypes were used to generate the VCF file containing all the variants in the second *in silico* haplotype (termed as Hap2).
(2) The two VCF files were used as inputs to generate two *in silico* donor genomes by *SimuG* (Yue and Liti, 2019).
(3) *ART* simulator (Huang et al., 2012) was employed to generate two 20× coverage data sets with both of the two *in silico* donor genomes, respectively, and they were merged as a 40× coverage data set for diploid genome. Two such data sets with various read lengths (2 × 150 bp and 2 × 250 bp) and same insert size (i.e., 500 bp) were generated. Refer to the Supplementary Materials for used command lines for the simulation.

### Real Data Sets for Benchmark

Two data sets produced by the Illumina platforms from the well-studied individual HG002, the son of the so-called Ashkenazi trio in GIAB, were employed to assess the ability of Psi-caller on real data sets. The data sets are paired end with different read lengths (2 × 148 bp and 2 × 250 reads, respectively), downloaded from the FTP server of GIAB (refer to Supplementary Materials). Moreover, hs37d5 was used as reference in the benchmark, and the high confidence SNV/INDEL benchmark set of HG002 provided by GIAB as ground truth callset, which has 3,452,896 SNVs and 585,700 Indels (with PASS filter tag). The download links are in the Supplementary Materials.

### Evaluation Metrics

The submodule *vcfeval* of RTG tool (v3.11) was used to evaluate the results of variant callers by three metrics, i.e., Precision, Recall, and F1 score, which are defined as follows:

(6) $$\begin{array}{c} P\,r\,e\,c\,i\,s\,i\,o\,n=\frac{T\,P\,s}{T\,P\,s+F\,P\,s} \end{array}$$

(7) $$\begin{array}{c} R\,e\,c\,a\,l\,l=\frac{T\,P\,s}{T\,P\,s+F\,N\,s} \end{array}$$

(8) $$\begin{array}{c} \mathrm{F1}=\frac{2\times P\,r\,e\,c\,i\,s\,i\,o\,n*R\,e\,c\,a\,l\,l}{P\,r\,e\,c\,i\,s\,i\,o\,n+R\,e\,c\,a\,l\,l} \end{array}$$

where TP, FP, and FN are true positives, false positives, and false negatives, respectively. FP are defined as variants existing in the GIAB data set that are also identified as a variant by callers, but with discrepant variant type, alternative allele. FN are defined as the variants existing in the GIAB data set but identified as non-variant by callers. F1 score is the harmonic mean of the precision and recall.

## Results

### Simulation Benchmarks

We assessed the baseline sensitivity and accuracy of Psi-caller with the two 40× simulated data sets at first. The reads were aligned to human reference genome (version: hs37d5) by BWA-MEM (v0.7.15) and sorted by Samtools beforehand. Three state-of-the-art variant callers, i.e., GATK HaplotypeCaller (v4.1.9.0), FreeBayes (v1.3.5), and Clair (v2.1.1), were implemented on the simulated data sets for comparison. The yields of Psi-caller and the three state-of-the-art callers are shown in Table 1. SNVs and Indels were assessed separately.

**TABLE 1:** Results of simulation benchmarks for various callers.

Data set	Method	SNV			INDEL		
		Precision	Recall	F1	Precision	Recall	F1
148PE	GATK	0.9998	0.9980	**0.9988**	0.9971	0.9107	**0.9520**
	FreeBayes	0.9995	0.9674	0.9832	0.9979	0.8624	0.9252
	Clair	0.9986	0.9976	0.9981	0.9809	0.8979	0.9376
	Psi-caller	0.9991	0.9981	0.9986	0.9943	0.9042	0.9471
250PE	GATK	0.9998	0.9980	**0.9988**	0.9971	0.9108	**0.9520**
	FreeBayes	0.9992	0.9675	0.9831	0.9988	0.8621	0.9254
	Clair	0.9983	0.9974	0.9978	0.9815	0.8987	0.9383
	Psi-caller	0.9993	0.9981	**0.9988**	0.9953	0.9051	0.9481

*The bold values mean the best F1 scores in the two data sets respectively.*

All the four callers achieved high and close precisions in SNV calling. The sensitivities and F1 scores of Psi-caller and GATK are close to each other for SNVs. For FreeBayes, its sensitivity is about 3% lower than that of Psi-caller and GATK. This indicates that there could be some intrinsic drawbacks in the design and implementation of the Bayesian statistics method employed by FreeBayes so that some SNVs were missed. Moreover, the yields of Clair are also slightly lower than that of Psi-caller, especially caused by lower precisions. This could be due to potential overfitting as deep learning-based methods are used.

A similar trend was observed in Indel calling. The yields of Psi-caller and GATK are also higher than that of Clair and FreeBayes. More precisely, GATK achieved the highest F1 scores (95.20 and 95.20%) and outperformed Psi-caller by 0.5 and 0.4% for PE150 and PE250, respectively. This is mainly due to the relatively lower sensitivity of Psi-caller, especially the ability to detect Indels in repeat-rich loci (refer to “Discussion” section for more details), although the precisions of Psi-caller and GATK are close. Both Clair and FreeBayes had lower F1 scores; however, they had different shortcomings. Clair had obviously lower precisions, although its sensitivities were also slightly lower than that of Psi-caller and GATK, suggesting that Indel calling is still a difficult task to this deep learning-based method. The precision of FreeBayes is high; however, its sensitivity is much lower, similar to that of SNV calling.

### Real Sequencing Data Benchmark

Psi-caller, GATK, FreeBayes, and Clair were further implemented on two real sequencing data sets produced by Illumina platforms (2 × 148 bp and 2 × 250 reads, refer to “Materials” section for more details) to assess their ability. The total numbers of variant calls are in the Supplementary Table 1. We assessed the number of calls produced by various approaches as well as their consistency at first. Psi-caller on average detected 3.8 million SNVs and 0.88 million Indels for the two data sets (“PASS” filter tag on auto chromosomes only). Meanwhile, the numbers of SNV calls produced by GATK and FreeBayes are close to that of Psi-caller. However, there are higher numbers of SNVs in the callset of Clair, i.e., more than 4 million for both of the two data sets. For Indels, the four approaches had more divergent numbers of calls. Clair had much higher numbers of calls (>900K), while it is much lower for that of FreeBayes (around 700K). Psi-caller and GATK had similar numbers of calls for the 2 × 148 bp data set; however, Psi-caller detected nearly 70K more Indels than GATK on the 2 × 250 bp data set (879K vs. 811K).

**FIGURE 2:**
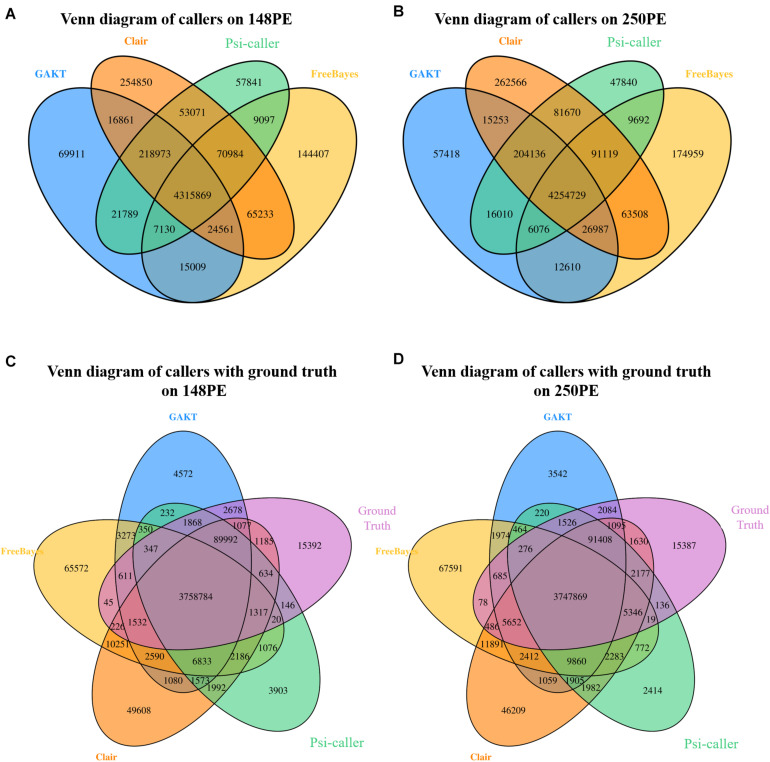
**(A,B)** The Venn diagram of variant calls produced by different tools on Illumina 148PE and 250PE reads, respectively; **(C,D)** The Venn diagram of variant calls produced by different tools and ground truth on Illumina 148PE and 250PE reads, respectively.

Venn diagrams of the callsets (along the whole genome) are shown in Figures 2A,B, which helps in investigating the consistency of the detected variants. Mainly, 4,315,869 and 4,254,729 SNV and Indel calls were commonly identified by all the callers on the two real sequencing data, respectively. Moreover, Psi-caller had the least number of unique calls than other tools (57,841 for 148PE and 47,840 for 250PE), meanwhile, GATK also had comparable unique calls. We also investigated the intersect with the ground truth callset in the high confidence region (Figures 2C,D). Psi-caller is still the caller having least unique calls (3,903 and 2,414 for the two data sets, respectively), indicating that there are less false positives in its callsets.

**FIGURE 3:**
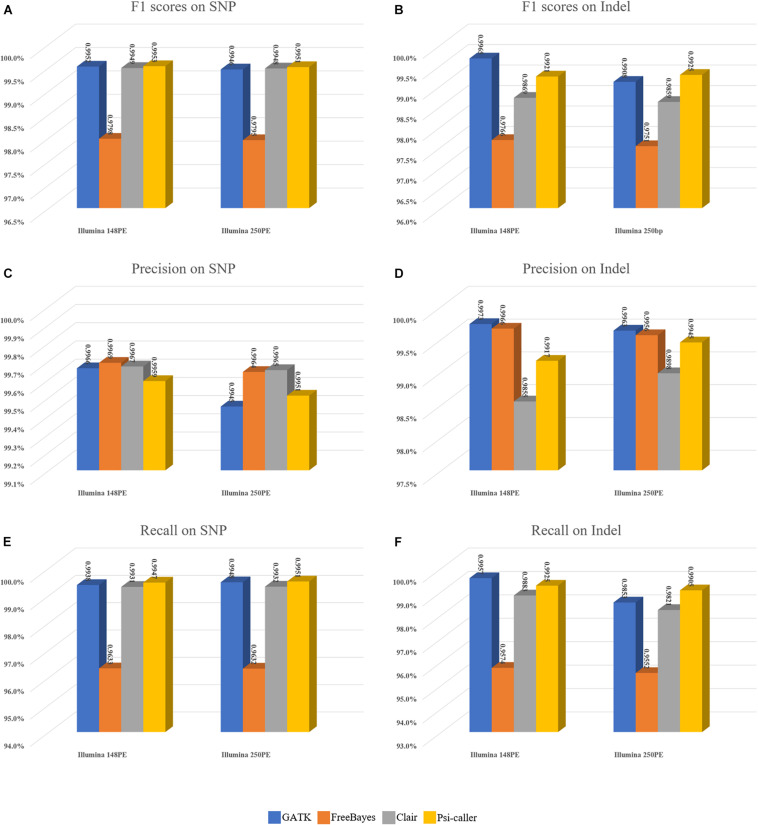
Benchmark results of the variant callers on real sequencing data sets. **(A,B)** F1 scores on SNVs and Indels, respectively. **(C,D)** Precisions on SNVs and Indels, respectively. **(E,F)** Recalls on SNVs and Indels, respectively.

The callsets of the four approaches were then compared with the ground truth callset by *vcfeval* module of the RTG tool (Figure 3). Moreover, Venn diagrams of the callsets produced by the four callers (for high confidence regions only) as well as the ground truth callsets are in Supplementary Figure 4 (SNVs and Indels are provided separately) for more detailed information. For SNV calling, Psi-caller achieved the highest precision and F1 score, 99.47 and 99.51% on the two data sets in absolute terms, respectively. Meanwhile, the F1 scores of GATK and Clair are close to that of Psi-caller (i.e., GATK: 99.52 and 99.46%, Clair: 99.49 and 99.48% for the PE148 and 250PE data sets, respectively). The F1 scores of FreeBayes are lower (97.98 and 97.95% for the PE148 and 250PE data sets, respectively), mainly due to its much lower recall rates, although its precision rates are higher or comparable to other callers. This trend is similar to that of the simulated data sets. It is also observed from the Venn diagrams (Supplementary Figures 4A,C) that Psi-caller still has the least numbers of unique calls.

For Indel calling, Psi-caller and GATK are the best two callers with relatively higher F1 scores (all of them are >99%), outperforming Clair and FreeBayes by 1 to 2%. For PE150 data, GATK is the best caller whose F1 score is slightly higher (by 0.44%) than that of Psi-caller; meanwhile, Psi-caller outperformed GATK by 0.17% for the PE250 data set. The performance of Indel is similar to SNVs for FreeBayes, i.e., low recalls with comparable precisions. The F1 scores of Clair are lower than those of Psi-caller and GATK, mainly due to its lowest precisions, while its recalls were only higher than that of FreeBayes as well. We further investigated the callsets of Psi-caller and GATK and found that GATK has better ability to handle clipped reads with its local assembly based approach, so that it enables to detect more Indels with shorter reads (148PE). Meanwhile, the POA-based method of Psi-caller is more sensitive to the Indel signatures implied by alignment details (CIGARs), so that it achieved higher F1 score for the longer reads (250PE). The Venn diagrams indicate that Psi-caller and GATK had comparable least numbers of unique Indels, respectively, which also partially indicates their similar ability to the detection of Indels.

### Performance of Psi-Caller

**FIGURE 4:**
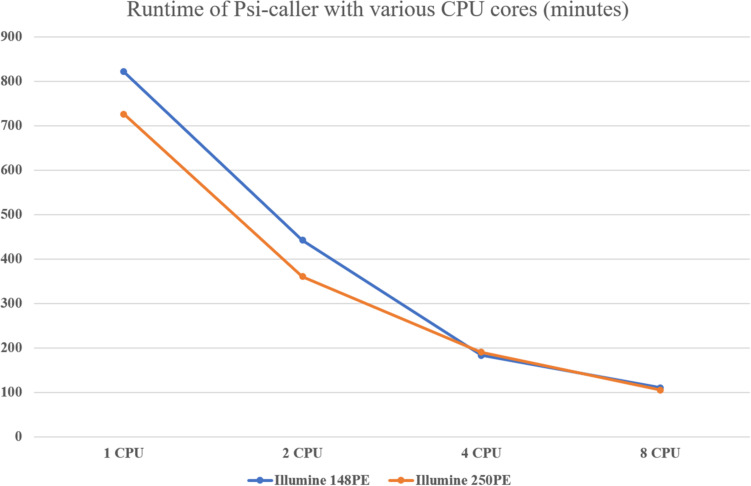
Performance of Psi-caller using different number of threads.

We assessed the speed of the callers since time cost is also a major concern for the analysis of large-scale data sets or time-sensitive tasks. The result is given in Table 2 and Figure 4. With eight CPU cores, the speed of Psi-caller is tens of times faster than GATK, a few times faster than FreeBayes and slightly outperformed Clair as well. It is also worth noting that Clair is a deep learning-based approach that needs time-consuming training process, although it can be done in advance, which can be downloaded from http://www.bio8.cs.hku.hk/clair_models/illumina/12345.tar. However, Psi-caller does not need training as well as any extra requirement on hardware such as GPUs. We also benchmark the speedup of Psi-caller with different CPU cores (Figure 4); the results show that Psi-caller has high scaling performance, i.e., it achieved a quasi-linear speedup with the number CPU cores and its clock time was greatly reduced. Considering its good sensitivity, precision, and high speed, Psi-caller is suited to handle large-scale short-read sequencing data sets, which has potentials to many large-scale genomics studies.

**TABLE 2:** Performance of the benchmarked variant callers.

Running time (min)	GATK	FreeBayes	Clair	Psi-caller
148PE	1244	260	117	**105**
250PE	1293	313	124	**101**

The high-speed feature of Psi-caller derives from both of its designs and implementations. Mainly, SKSV divides the candidate variant sites into three categories, and the vast majority of candidates belong to HCs (details shown in Supplementary Table 2), which greatly speed up variant calling using the direct binomial model. In addition, the POA-based method also helps improve the speed compared to local assembly. Moreover, the block division-based subtask implementation also helps Psi-caller to achieve outstanding scaling performance. Psi-caller uses Pypy (Rigo Aea, 2018), a Just-In-Time (JIT) compiler, other than the native python interpreter to implement the operations of subtasks. Because of the existence of Global Interpreter Lock (GIL), the performance cannot be improved by using multiple threading when sample processing. However, the JIT built-in compiler and synchronous programming of Pypy greatly reduce run-time for large programs.

## Discussion

The rapid development of HTS technologies is promising to comprehensively discover genomic variants of various populations. However, variant calling is still a computationally intensive task whose cost is non-neglectable. Moreover, cutting-edge genomics studies also have high requirements on the sensitivity and accuracy of variant calling. Herein, we propose Psi-caller, a novel lightweight short read-based variant calling approach, as a solution to this important open problem. Mainly, we show how to achieve higher yield and performance in variant calling with tailored candidate variant sites recognition, consensus sequence reconstruction, and variant calling and genotyping methods for various kinds of candidate variant sites. Benchmarks on simulated and real data sets suggest three features of Psi-caller as follows:

(1) Psi-caller uses simple statistical rules to recognize candidate variant sites from pileup alignments and divides the candidate variant sites into three categories, i.e., high confidence candidates (termed as HCs), low confidence candidates (termed as LCs), and candidates in tandem repeat regions (termed as TRCs), according to the complexity and location of the sites with strict definitions and thresholds. Two examples are shown in Supplementary Figures 2, 3. They show candidate variant sites with homogeneous and evident signatures and complex signatures, respectively.
(2) Psi-caller employs various approaches, i.e., direct binomial model, POA, and de Bruijn graph-based local assembly to call and genotype SNVs and Indels in HCs, LCs, and TRCs, respectively. An example is shown in Supplementary Figure 5. The specifically designed POA and local assembly based methods enable complicated signatures of SNV and Indels to be well handled.
(3) Psi-caller has outstanding speed with the help of subtask division strategy and JIT compiler in data processing. This is suited to modern high-performance computing cluster and also scalable to large-scale data analysis tasks.

The major advantage of Psi-caller is its outstanding speed, which is tens of times faster than GATK. Meanwhile, benchmarks on both of simulated and real sequencing data sets demonstrate that Psi-caller has equal or higher good sensitivity and accuracy to state-of-the-art variant callers. For SNV calling, Psi-caller achieved comparable or higher F1 scores, and it also had the least number of unique calls compared to other benchmarked state-of-the-art callers. This indicates that Psi-caller is able to produce accurate and reliable callsets. For Indel calling, Psi-caller and GATK are the best two callers with relatively higher F1 scores in the benchmark. In PE150, Psi-caller is the best runner-up, but in PE250 real sequencing data set, Psi-caller outperform GATK by 0.17%.

However, Psi-caller still has a few shortcomings like the relatively lower recalls on Indels. We investigated the detailed intermediate results of Psi-caller and observed the following four issues, which could be important to further improve the sensitivity of this approach.

(1) For some Indels in the ground truth callset, Psi-caller fails to detect them due to that there is no candidate variant site recognized from the input short-read alignments. Most of them are located in short tandem repeat regions. It is still a bottleneck for short read aligners to produce accurate alignments in such regions, so that the detection of variant is affected. An example is in Supplementary Figure 6. In this case, the variant is in a typical short tandem repeat region; however, no evident signature is implied by the alignment of the reads around the variant site.
(2) For some Indels, Psi-caller can recognize the corresponding variant site as candidates, although they are also in repeat-rich regions. However, the assembly method produces false-positive or false-negative variant calls due to the failure of local assembly, mainly caused by the high repetitiveness of local genomic sequences. The example displayed in Supplementary Figure 7 illustrates that only some of candidates can be detected as variants due the power of assembly or fewer supporting alignments.
(3) Some of Indels are recognized by several nearby false positives due to the scoring system of KSW2. An example in Supplementary Figure 8, which obtains incorrect variants using a small mismatch penalty score, which causes SKW2 to regard alignment containing one deletion and two mismatches (i.e., SNVs) as the best result. However, it is still a difficult open problem to design a highly effective scoring system to fit all kinds of variants; hence, it could be a potential solution to use specifically designed scoring system for some difficult regions.
(4) Some Indels in low confidence sites cannot be detected by abPOA due to the ratio of supporting reads ratio and the number of reads to recognize the contigs carrying alternative alleles.

In order to improve the sensitivity of Indels, more advanced recognition and filtration strategies for candidate variant sites and more accurate partial order alignment and local assembly algorithm for detecting variants should be employed in the future works.

## Data Availability Statement

The original contributions presented in the study are included in the article/Supplementary Material, further inquiries can be directed to the corresponding authors.

## Author Contributions

YL, TJ, and BL designed the method. YL implemented the method. All authors performed the analysis, wrote the manuscript, read, and approved the final manuscript.

## Conflict of Interest

The authors declare that the research was conducted in the absence of any commercial or financial relationships that could be construed as a potential conflict of interest.

## Publisher’s Note

All claims expressed in this article are solely those of the authors and do not necessarily represent those of their affiliated organizations, or those of the publisher, the editors and the reviewers. Any product that may be evaluated in this article, or claim that may be made by its manufacturer, is not guaranteed or endorsed by the publisher.
