# Supplementary material for: Psi-Caller: A Lightweight Short Read-Based Variant Caller With High Speed and Accuracy
Source: Front Cell Dev Biol. 2021 Aug 13;9:731424. doi: 10.3389/fcell.2021.731424 (PMC8414796; doi:10.3389/fcell.2021.731424)
Supplement: Supplementary file 1 [file Data_Sheet_1.PDF]

Supplementary Material

1 Supplementary Data

1.1 Implementation of simulation

The ground truth variants for simulation generated by high confidence variants of HG001 sample from GIAB, which can be downloaded from:

https://ftp-trace.ncbi.nlm.nih.gov/ReferenceSamples/giab/release/NA12878_HG001/latest/GRCh37/

The following four steps with corresponding commands are the implementation for simulations:

1) Extract variant whose genotype equal ‘0|1’ and ‘1|1’ to generate the VCF file containing all variants in the first *in silico* haplotype (termed as Hap1). Similarly, genotype with ‘1|0’ and ‘1|1’ are used to generate the VCF file containing all the variants in the second *in silico* haplotype (termed as Hap2).

```
cat <(bcftools view -h hg001.vcf.gz --no-version) <(bcftools view -H --no-version hg001.vcf.gz | awk
'{split($10,a,":"); gt=a[1]; if(gt=="0|1" || gt == "1|1") print $0}') | bgzip -c >hg001.h1.vcf.gz
```

```
cat <(bcftools view -h hg001.vcf.gz --no-version) <(bcftools view -H --no-version hg001.vcf.gz | awk
'{split($10,a,":"); gt=a[1]; if(gt=="1|0" || gt == "1|1") print $0}') | bgzip -c >hg001.h2.vcf.gz
```

2) Split the Hap1 and Hap2 into separated SNV and INDEL VCF files as input.

```
Bcftools view -v SNVs hg001.h1.vcf.gz -Oz -o hg001.h1_SNV.vcf.gz
Bcftools view -v SNVs hg001.h2.vcf.gz -Oz -o hg001.h2_SNV.vcf.gz
Bcftools view -v Indels hg001.h1.vcf.gz -Oz -o hg001.h1_Indel.vcf.gz
Bcftools view -v Indels hg001.h2.vcf.gz -Oz -o hg001.h2_Indel.vcf.gz
```

3) Use the two VCF files as input to generate two *in silico* donor genomes by *SimuG*[1].

```
perl simuG.pl -refseq /home/ydliu/MLSV/data/REF/hs37d5_2.fa -SNV_vcf hg001.h1_SNV.vcf.gz -Indel_vcf
hg001.h1_Indel.vcf.gz -prefix sim_h1
perl simuG.pl -refseq /home/ydliu/MLSV/data/REF/hs37d5_2.fa -SNV_vcf hg001.h2_SNV.vcf.gz -Indel_vcf
hg001.h2_Indel.vcf.gz -prefix sim_h2
```

4) Employ *ART* simulator[2] to generate two 15x coverage datasets with both of the two *in silico* donor genomes respectively and merge them as a 30x coverage dataset for diploid genome.

```
art_illumina -ss HS25 -i sim_h1.fa -p -l 150 -f 15 -m 500 -s 10 -o h1
art_illumina -ss HS25 -i sim_h2.fa -p -l 150 -f 15 -m 500 -s 10 -o h2
```

1.2 Implementation of benchmark

We implemented the benchmark on a server with AMD Ryzen 3950X CPU and 120GB RAM, running Linux Ubuntu 16.04.

The real HG002 Illumina PE150 reads was downloaded from: https://ftp-trace.ncbi.nlm.nih.gov/giab/ftp/data/AshkenazimTrio/HG002_NA24385_son/NIST_HiSeq_HG002_Homogeneity-10953946/

The real HG002 Illumina PE250 reads was downloaded from: https://ftp-trace.ncbi.nlm.nih.gov/ReferenceSamples/giab/data/AshkenazimTrio/HG002_NA24385_son/NIST_Illumina_2x250bps/

The GIAB ground truth set and corresponding high confidence region set were downloaded from: https://ftp-trace.ncbi.nlm.nih.gov/ReferenceSamples/giab/release/AshkenazimTrio/HG002_NA24385_son/latest/GRCh37/

RTG tools (v3.11) was used to assess the precision, recall, and F1 score against the GIAB SNV/Indel benchmark set[3].

1.2.1 Command lines for performing BAW (version 0.7.17)

Genome indexing:

```
bwa index /path/hs37d5.fa -p
```

Read alignment:

```
bwa mem -t 12 -R "@RG\tID:hg2\tSM:hg002" /path/hs37d5.fa read1.fq read2.fq -o aln.sam
```

1.2.2 Command lines for SAMtools processing (version 1.11)

Alignment viewing, sorting and indexing:

```
samtools view -bS aln.sam | samtools sort -O bam -o aln.bam && samtools index aln.bam
```

1.2.3 Command lines for performing variant callers

GATK HaplotypeCaller (version 4.1.9.0)

```
gatk HaplotypeCaller -I aln.bam -R /path/hs37d5.fa -O gatk.vcf --native-pair-hmm-threads 8
```

FreeBayes (version 1.3.5)

```
freebayes -f /path/hs37d5.fa aln.bam -C 5 >freebayes.vcf
```

For parallelization:

```
freebayes-parallel <(fasta_generate_regions.py /path/hs37d5.fa.fai 10000000) 8 -f /path/hs37d5.fa aln.bam > freebayes.vcf
```

Clair (version 2.1.1)

```
clair.py callVarBamParallel --chkpnt_fn /ClairPath/model/illumina/model --ref_fn /path/hs37d5_2.fa --bam_fn aln.bam --threshold 0.1 --sampleName HG002 --output_prefix workspace/var > task.sh

cat task.sh | parallel -j 8

vcfcat workspace/var.*.vcf | bcftools sort -m 2G | bgzip > clair.vcf.gz
```

Psi-caller (version 1.0)

```
python generate_task.py aln.bam /path/hs37d5.fa workspace/ > commands.sh

cat commands.sh | parallel -j 8

bash post_process.sh workspace/ Psi-caller.vcf
```

1.2.4 Command lines for benchmarking variant callers using rtgtools (version 3.11)

```
rtg format hs37d5.fa -o hs37d5.sdf/;

rtg vcfeval  -b ground_truth.vcf.gz -e high_confidence.bed -t hs37d5.sdf/ -o bench/ -f QUAL -c {caller}.vcf.gz --squash-ploidy
```

2 Supplementary Figures and Tables

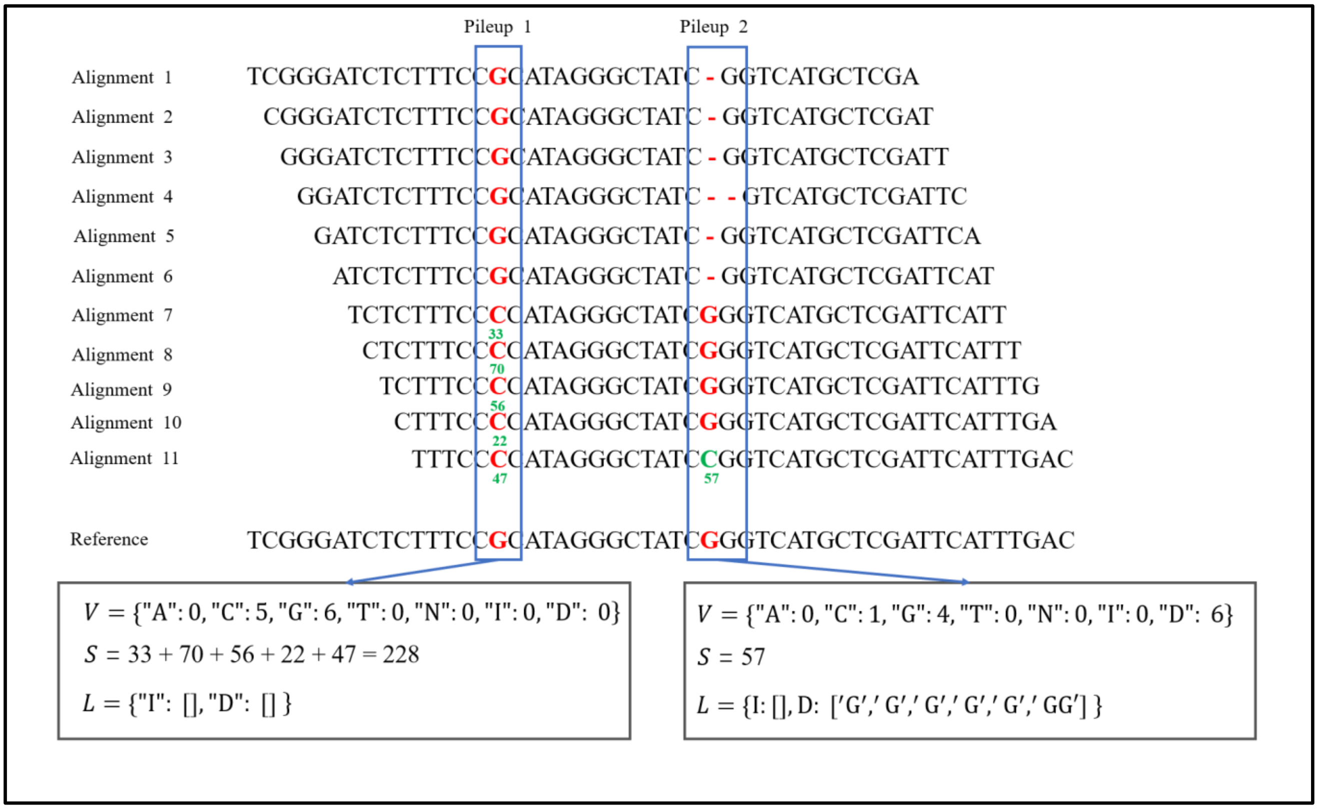

Supplementary Figure 1. An schematic illustration of alignment pileup. Psi-caller pileups 11 alignments and recognizes two variant candidate sites and record the detailed information in triple consisting of three variables. The pileuped sequence with variants are recovered by CIGARs. The first candidate is a heterozygous SNV whose alternative allele is ‘C’ and the total quality score (green number under the allele base) is summed as 228. The second candidate is a heterozygous Indel with 6 supporting alignments (one for ‘GG’ and five for ‘G’ allele sequence).

A

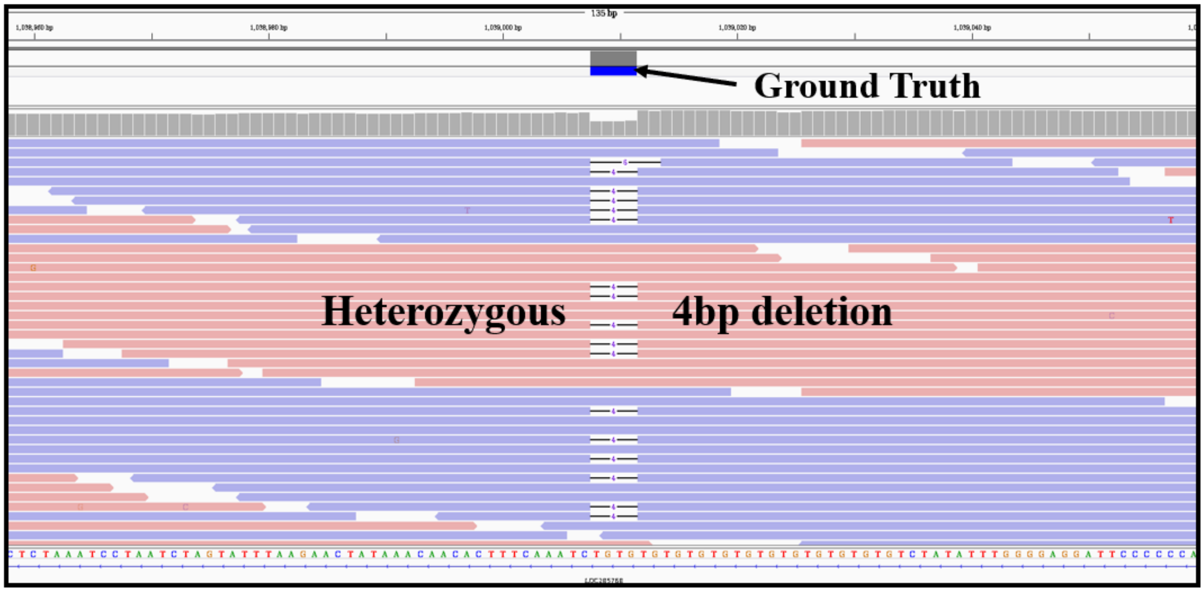

B

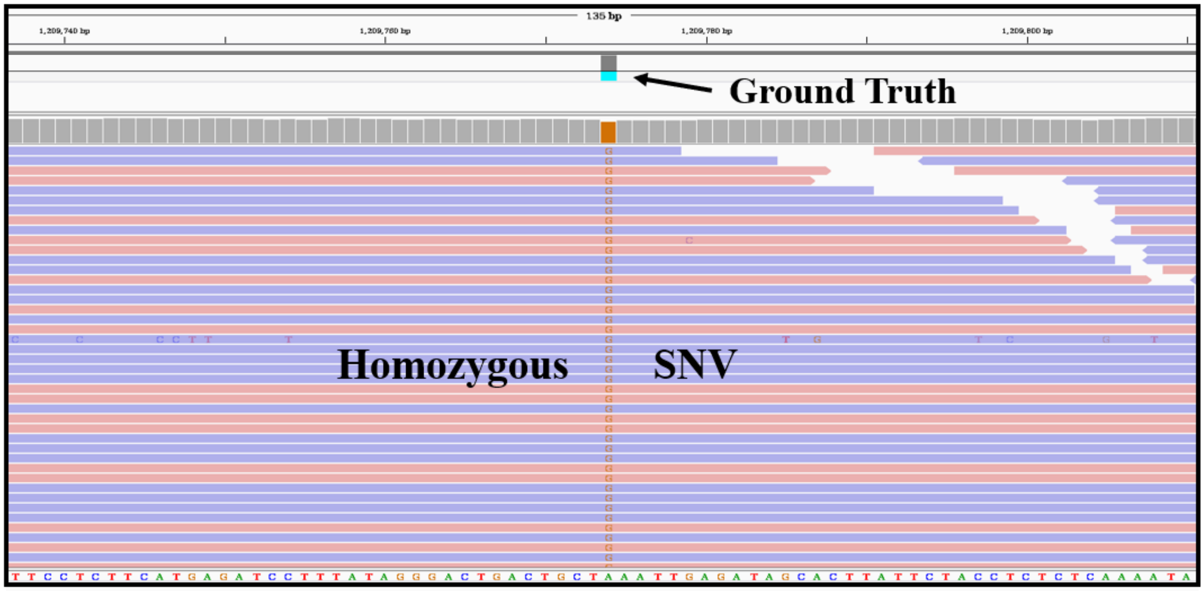

Supplementary Figure 2. Examples for candidate site with homogeneous and evident signatures. A) A heterozygous 4bp deletion in the ground truth (chr6:1,039,007 of HG002 sample), and the signatures in CIGAR is homogeneous. Alignments nearby are pretty and no other candidate site can be recognized. B) A homozygous SNV (chr6:1,209,774 of HG002 sample) with obvious and clean signatures.

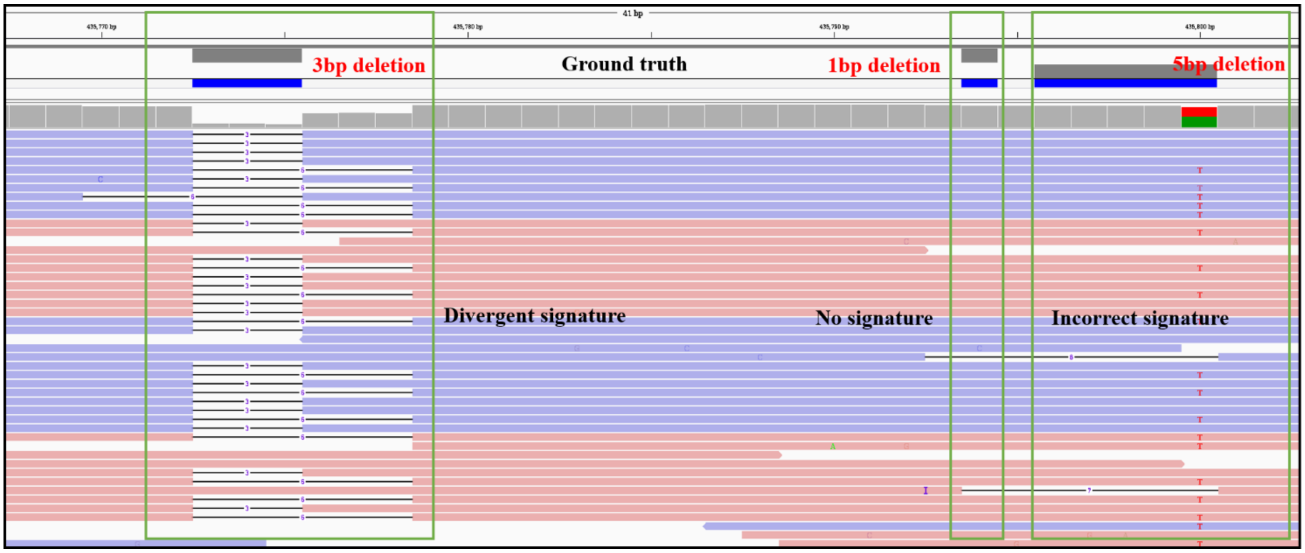

Supplementary Figure 3. An example for candidate site with complex signatures. In the ground truth, there are three heterozygous variants on chr2, i.e. 3bp deletion at 435,772, 1bp deletion at 435,793 and 5bp deletion at 435,795. But the snapshot of the alignment in Integrative Genomics Viewer (IGV)[4] shows complex signatures. At 435,772, there are two main deletion signatures, that is 3bp and 6bp deletion, which may regard as a heterozygous deletion with two different alleles. At 435,793 and 435,795, no signatures or incorrect signatures comparing to the ground truth.

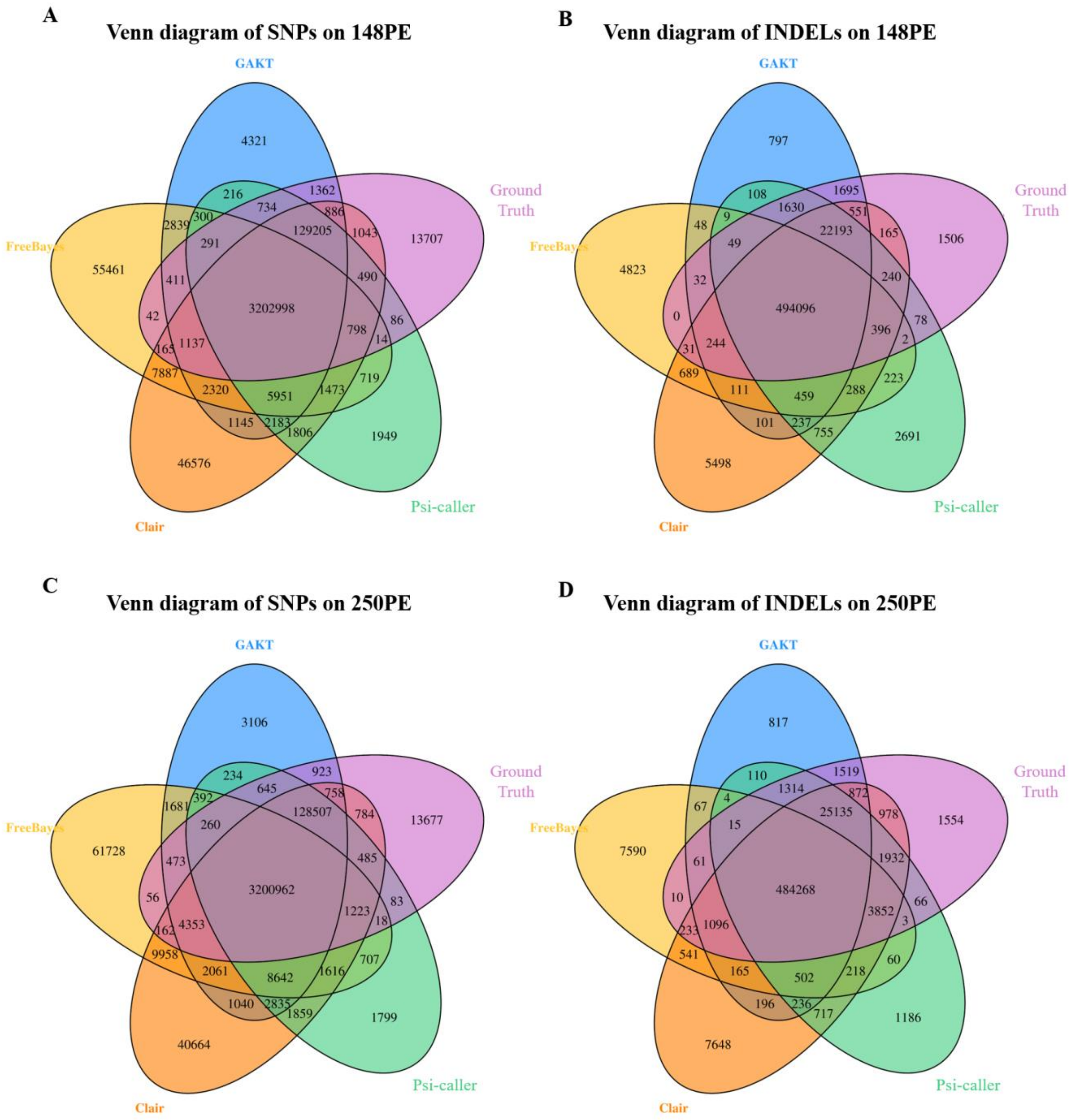

Supplementary Figure 4. A-B) The Venn diagram of SNV and Indel calls produced by different tools and the ground truth on Illumina 148 PE reads respectively; C-D) The Venn diagram of SNV and Indel calls produced by different tools and the ground truth on Illumina 250 PE reads.

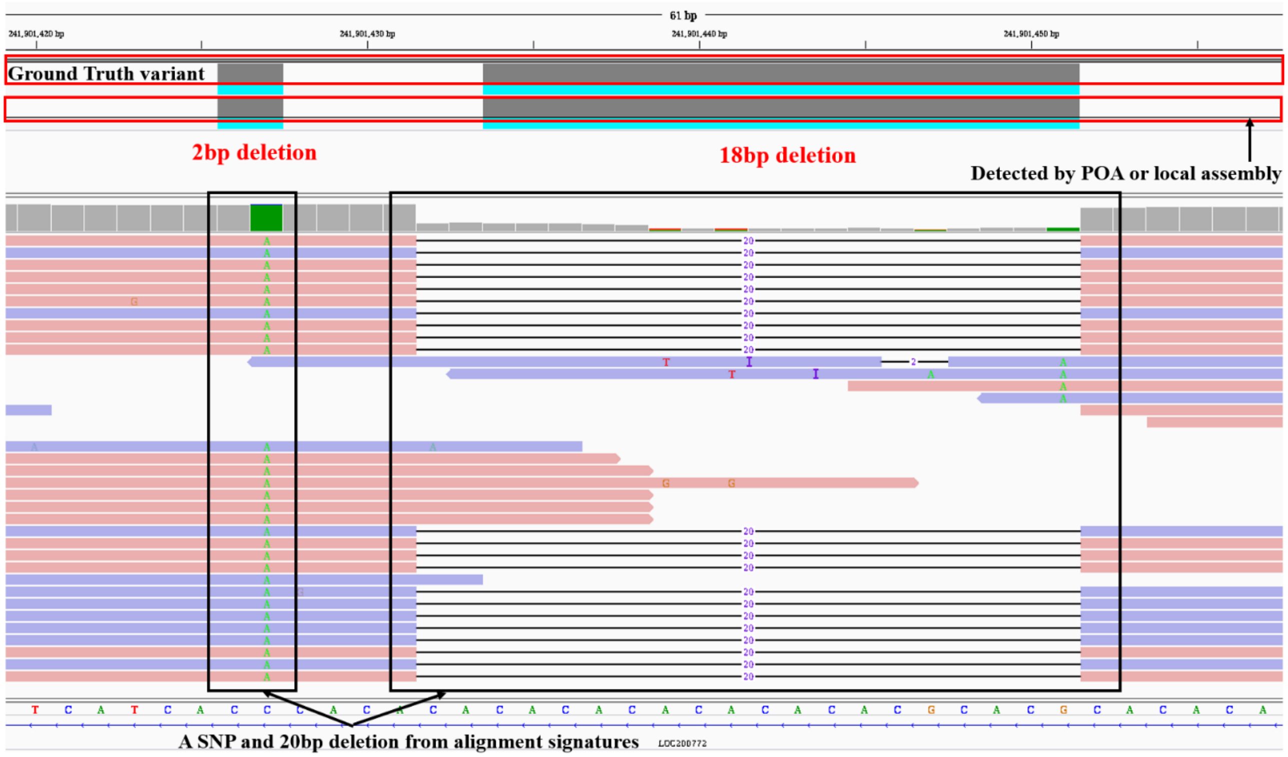

Supplementary Figure 5. An example of variant calling using POA or local assembly to get genuine variants. There are two homozygous variants on chr2, i.e. 2bp deletion at 241,901,425, 18bp deletion at 241,901,433 in the ground truth. However, from the pileup alignments, two homogeneous and evident signatures are found which seeming as a SNV at 241,901,427 and a 20bp deletion at 241,901,431. In fact, the distance of the two candidate sites less than 10bp, and located in a tandem short repeat region with "ACACAC" repeats (according to the annotation on human reference genome at: https://github.com/PacificBiosciences/pbsv/tree/master/annotations), short-read alignments are incorrect. With the using POA or local assembly methods, two corresponding right variants can be found.

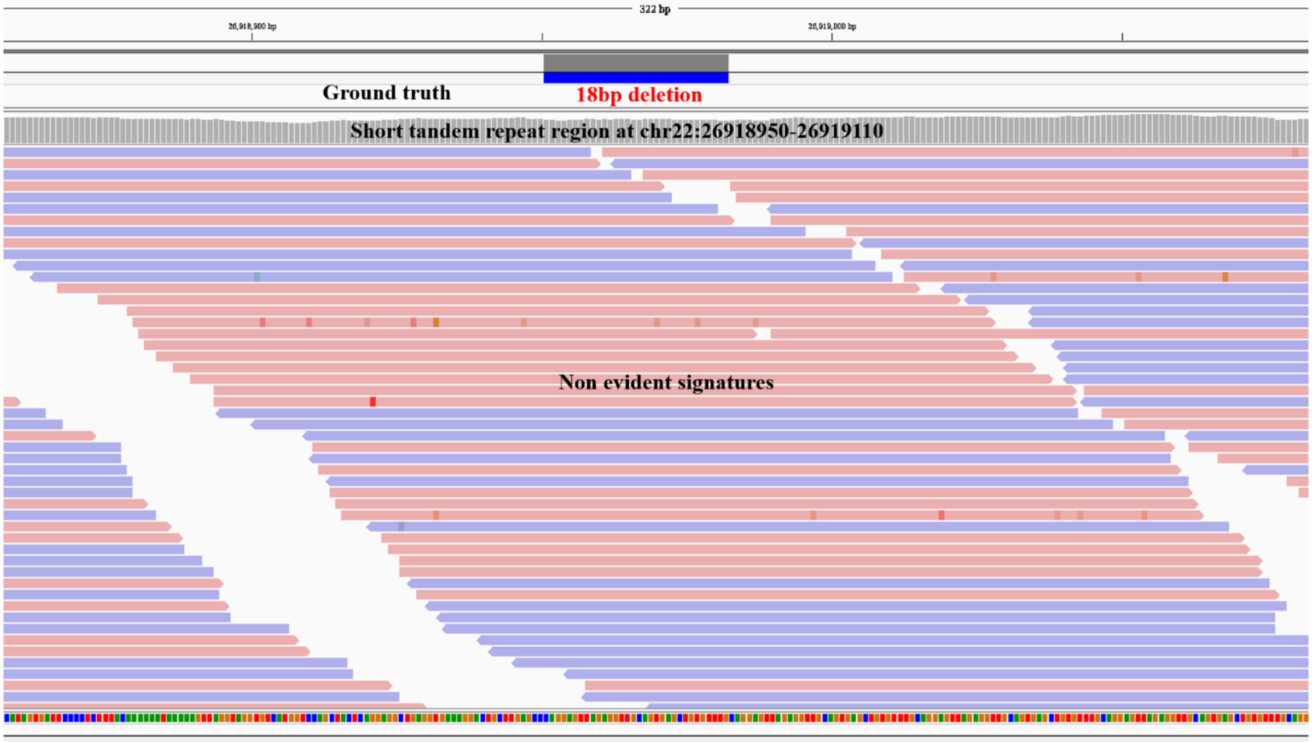

Supplementary Figure 6. An example ground truth Indel without any evident signatures. In the ground truth callset, there is a 18bp deletion at chr22: 26,918,950, which located in a short tandem repeat range chr22: 26,918,950-26,919,110. From the short-read alignments, no evident signatures supporting the variant can be found in the “Candidate recognition” step of Psi-caller, which causes the missing in the final callset of Psi-caller.

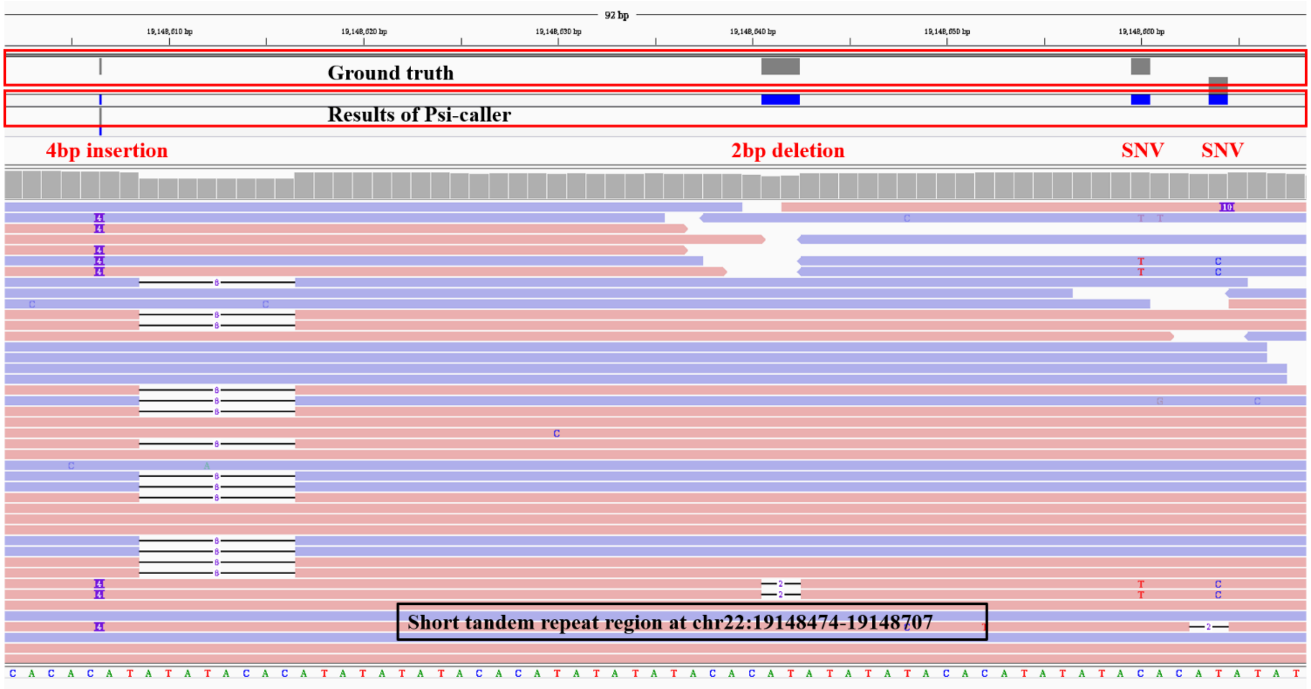

Supplementary Figure 7. An example of ground truth can't be detected due to false assembly. In the ground truth callset, there are four variants located in a short tandem repeat range chr22:19,148,474-19,148,707, that is a 4bp insertion at chr22:19,148,606, a 2bp deletion at chr22:19,148,640, and two SNVs at chr22:19,148,660 and chr22:19,148,664 respectively. When applying a local assembly approach, only the 4bp insertion can be detected correctly. Mainly, there exist lots of "AT" and "AC" repeats and "C->T" and "T->C" substitutions, the assembly seems to be insusceptible with such complex circumstances.

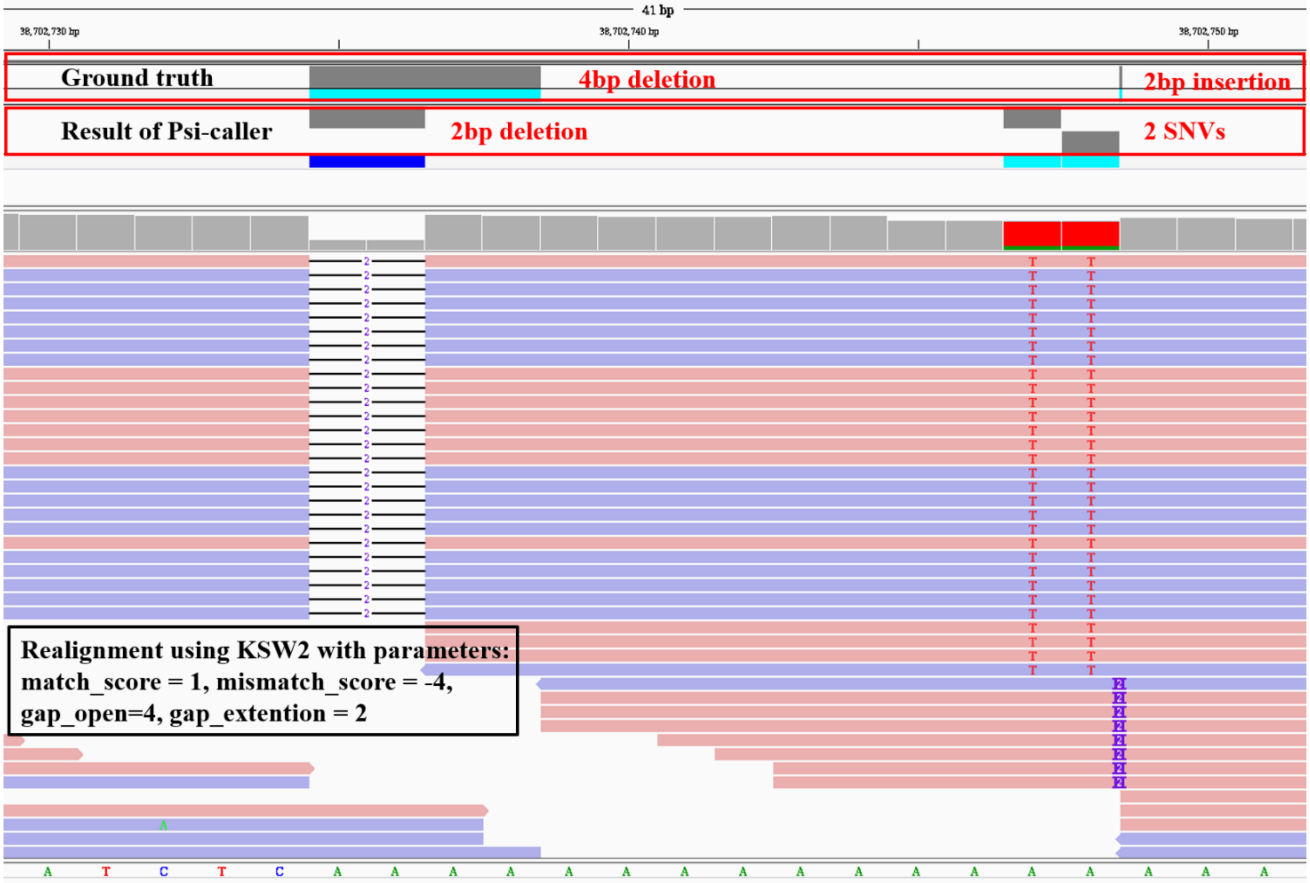

Supplementary Figure 8. An example of false positives caused by the scoring system KSW2. One 4bp deletion at chr22: 38,702,734 and one 2bp insertion at chr22:38,702,748 in the ground truth callset are recognized by a 2bp deletion chr22: 38,702,734 and 2 SNVs at chr22: 38,702,747 and chr22: 38,702,748 respectively. Actually, when increasing penalty score to -6 for mismatch (i.e. SNV), Psi-caller can output correct variants.

Supplementary Table 1. The statistical of variant calling

Method	PE148		PE250	
	# SNVs	# Indels	# SNVs	# Indels
**GATK**	3812304	883066	3786694	811013
**FreeBayes**	3758277	766309	3809370	713900
**Clair**	4066350	960119	4053834	951906
**Psi-caller**	3850661	888301	3838712	879476

Supplementary Table 2. The number of candidate variant sites in different categories

Datasets	# HCs	# LCs	# TRCs
**148 PE**	4115873	188192	570829
**250 PE**	4088794	234918	572752

Reference

1. Yue JX, Liti GN. simuG: a general-purpose genome simulator, Bioinformatics 2019;35:4442-4444.

2. Huang W, Li L, Myers JR et al. ART: a next-generation sequencing read simulator 2012;28:593-594.

3. Zook JM, Chapman B, Wang J et al. Integrating human sequence data sets provides a resource of benchmark SNP and Indel genotype calls, Nature Biotechnology 2014;32:246-251.

4. Robinson JT, Thorvaldsdóttir H, Winckler W et al. Integrative genomics viewer 2011;29:24-26.
